# Carotid Sinus Massage in Syncope Evaluation: A Nonspecific and Dubious Diagnostic Method

**DOI:** 10.5935/abc.20180114

**Published:** 2018-07

**Authors:** Tan Chen Wu, Denise T. Hachul, Francisco Carlos da Costa Darrieux, Maurício I. Scanavacca

**Affiliations:** Instituto do Coração (InCor) - Faculdade de Medicina da Universidade de São Paulo, São Paulo, SP - Brazil

**Keywords:** Syncope, Carotid Sinus / physiopathology, Accidental Falls, Aged, Hypotension

## Abstract

**Background:**

Carotid sinus hypersensitivity (CSH) is a frequent finding in the evaluation of syncope. However, its significance in the clinical setting is still dubious. A new criterion was proposed by Solari et al. with a symptomatic systolic blood pressure (SBP) cut-off value of ≤ 85 mmHg to refine the vasodepressor (VD) response diagnosis.

**Objective:**

To determine and compare the response to carotid sinus massage (CSM) in patients with and without syncope according to standard and proposed criteria.

**Methods:**

CSM was performed in 99 patients with and 66 patients without syncope. CSH was defined as cardioinhibitory (CI) for asystole ≥ 3 seconds, or as VD for SBP decrease ≥ 50 mmHg.

**Results:**

No differences in the hemodynamic responses were observed during CSM between the groups, with 24.2% and 25.8% CI, and 8.1% and 13.6% VD in the symptomatic and asymptomatic groups, respectively (p = 0.466). A p value < 0.050 was considered statistically significant. During the maneuvers, 45 (45.45%) and 34 (51.5%) patients in the symptomatic and asymptomatic groups achieved SBP below ≤ 85 mmHg. Symptoms were reported especially in those patients in whom CSM caused a SBP decrease to below 90 mmHg and/or asystole > 2.5 seconds, regardless of the pattern of response or the presence of previous syncope.

**Conclusion:**

The response to CSM in patients with and without syncope was similar; therefore, CSH may be an unspecific condition. Clinical correlation and other methods of evaluation, such as long-lasting ECG monitoring, may be necessary to confirm CSH as the cause of syncope.

## Introduction

Carotid sinus hypersensitivity (CSH), an age-related phenomenon, is rarely diagnosed in patients under the age of 50 years.^1^ It has been accepted as a cause of syncope and unexplained falls in the elderly, with prevalence as high as 45% in some reports.^2^

The clinical relevance of a positive response to carotid sinus massage (CSM) in patients with syncope is still controversial, in spite of the previous publications. Although the reported prevalence of CSH in patients with syncope is 23% to 41%,^3-8^ it has been described in 17% of normal subjects, in 20% of patients with cardiovascular disease, and in 38% of patients with severe carotid artery disease.^9-11^ Recently, some reports have proposed a modification of the diagnostic criterion according to hemodynamic findings during CSM,^12,13^ with a cut-off value of symptomatic systolic blood pressure (SBP) of ≤ 85 mmHg to determine a vasodepressor (VD) form, instead of the current definition of 50 mmHg SBP fall. To clarify the practical implications of CSM and CSH in syncope evaluation, this study was aimed at determining CSH prevalence and analyzing the patterns of the hemodynamic responses to CSM and symptoms in patients older than 50 years with and without symptoms of syncope or presyncope seen in a tertiary referral unit.

## Methods

The scientific and ethics committees of our institution approved this study. Written informed consent was obtained from each participant.

Patients aged 50 years or older with at least two episodes of syncope or presyncope in the previous year, referred to the Arrhythmia and Syncope Unit of the Instituto do Coração (InCor) – University of São Paulo Medical School Hospital were selected as the symptomatic group. The number of patients was determined by convenience sampling. Patients presenting with structural heart disease, such as dilated cardiomyopathy with a left ventricular ejection fraction ≤ 50%, moderate or significant valvular disease, myocardial infarction in the previous 6 months, unstable angina, stroke, carotid bruit or previously diagnosed carotid artery stenosis were excluded. Patients on chronic use of beta-blockers, digitalis, calcium channel blockers or alpha-methyldopa, who could not discontinue them, as well as patients with an artificial pacemaker, were also excluded.

For the asymptomatic group, 66 patients with no history of syncope or presyncope were selected from the geriatric outpatient clinic of the same institution. The exclusion criteria for the group were the same as those applied to the symptomatic group.

### Carotid sinus massage

Carotid sinus massage was performed from 1:00 pm to 5:00 pm. Cardiac medications, such as beta-blockers, calcium channel blockers (diltiazem and verapamil), digoxin and alpha-methyldopa, were discontinued 3 days before the procedure. All CSM were performed by the same physician. Continuous electrocardiogram and noninvasive, beat-to-beat blood pressure were recorded by digital photoplethysmography (Finapres Monitor® Ohmeda, USA)^14^ or a vascular unloading device (Task Force Monitor ® CNSystems Medizintechnik GmbH, Graz, Austria).^15-17^

Blood pressure was monitored in the first 3 minutes with the patient in the 70° upright position on a footplate-assisted tilt table to evaluate the presence of orthostatic hypotension (OH), which was defined as a postural drop in SBP of at least 20 mmHg or a drop in diastolic blood pressure (DBP) of at least 10 mmHg within the first 3 minutes of standing.^18^

Carotid sinus massage was performed for 5 seconds, in the 70° upright position after 5 minutes of standing, following the stabilization of blood pressure and heart rate, and at the point with the maximal carotid pulse on the anterior margin of the sternocleidomastoid muscle. Blood pressure and heart rate were monitored throughout. Right-sided CSM was followed by left-sided CSM (or vice versa) after at least 1 minute or as long as the heart rate and blood pressure values returned to baseline. The CSM was performed twice in each side to evaluate the reproducibility of the method. The sequence was completed even in the event of positivity of 1 massage. After each episode of CSM, patients were questioned about symptoms related to the maneuver. Cardioinhibitory (CI) CSH was defined as asystole of 3 seconds or more, and VD CSH was defined as a drop of 50 mmHg or more in SBP.^19^

Blood pressure was recorded continuously immediately before each CSM until it reached the lowest value recorded during or shortly after the maneuver. The magnitude of the blood pressure response was obtained by the difference between the baseline SBP and the minimum SBP during CSM (ΔSBP). Likewise, RR intervals were recorded, and the magnitude of heart rate response was given by the difference between the RR interval before CSM and the maximum RR interval during CSM (ΔRR).

### Statistical analysis

The data were analyzed by using Excel 2003 and SPSS software for Windows, version 15.0. The nominal measures are presented in absolute (n) and relative (%) frequencies, and numerical measurements are described as mean, standard deviation, median, minimum and maximum values. The clinical characteristics and responses to CSM (the order, result and symptoms associated with CSM) were compared between groups by using the chi-square test and the likelihood ratio test. The numerical measurements between the groups were summarized by descriptive statistics and compared by using Student *t* test, chi-square test for categorical data, and Mann-Whitney test for continuous data. Nonparametric tests were used in the absence of normally distributed data assumption (Kolmogorov-Smirnov test). The intraclass correlation coefficient was used to analyze the reproducibility of the CSM response. A p value of < 0.050 was considered statistically significant.

## Results

In the symptomatic group, almost all patients (93.9%) had syncope, with an average of 5.4 episodes (median - 3) in the year prior to evaluation. The baseline clinical characteristics of the 99 patients in the symptomatic group and the 66 patients in the asymptomatic group are shown in Table 1.

**Table 1:** Clinical characteristics of the symptomatic and asymptomatic groups.

Variable	Symptomatic (n = 99)	Asymptomatic (n = 66)	p
Age, mean ± sd (median) (minimum - maximum)	69.67 ± 10.26 (70) (50-93)	73.01 ± 9.68 (74) (52-92)	0.037
Male, n (%)	41 (41.4%)	23 (34.8)	0.396
Hypertension	73 (73.7%)	54 (81.8%)	0.227
Diabetes	13 (13.1)	20 (30.3)	0.007
Coronary artery disease	5 (5.1)	11(16.7)	0.014

chi-square test; sd: standard deviation.

**Figure 1:**
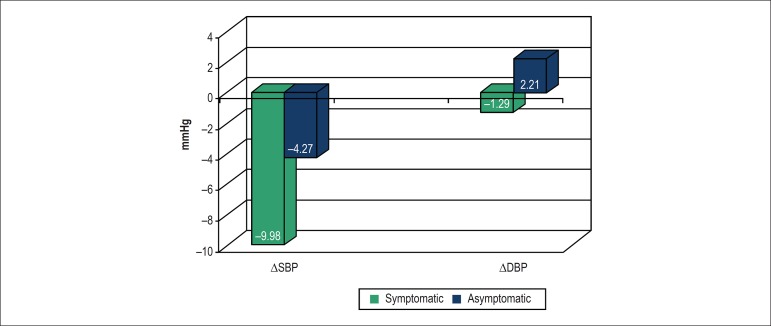
Magnitudes of the responses of systolic and diastolic blood pressure to a 70° tilt in the symptomatic and asymptomatic groups. Note there is significant fall in the systolic blood pressure (p < 0,001) and diastolic blood pressure (p = 0,001) in the symptomatic group compared with asymptomatic group.

Patients in the symptomatic group had the most significant decreases in blood pressure after being tilted to 70°. The mean SBP and DBP changes after orthostatic stimulus are shown in Figure 1. The symptomatic group had more occurrences of OH (29 patients, 29.2%), of whom, 19 patients met the diagnostic criterion of a SBP decrease ≥ 20 mmHg, and 10 additional patients met the criterion of a DBP decrease ≥ 10 mmHg. Only 8 patients (12.1%) in the asymptomatic group had a diagnosis of OH, which was due to decreased SBP in 7 of them (p = 0.014).

### Carotid sinus massage

**Figure 2:**
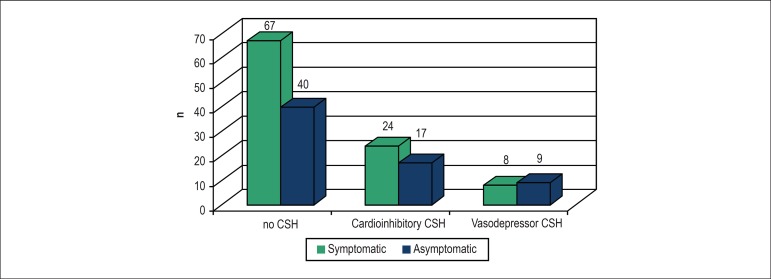
Results of carotid sinus massage according to the type of response obtained in the symptomatic and asymptomatic groups. CSH: carotid sinus hypersensitivity.

There was no difference between the groups in the responses obtained during CSM (p = 0.466) (Figure 2). The response to CSM was considered normal in 64.8% of patients in the entire sample, 67.7% in the symptomatic group and 60.6% in the asymptomatic group. Over 32% of the patients in both groups had an abnormal response to CSM, with predominance of CI responses.

Men had more abnormal responses to CSM compared to women (53.8% vs. 23.0%, p < 0.001). A predominance of CI response was also observed in men compared to women (43.1% vs. 13.0%). There was no significant difference in responses to CSM related to age. Likewise, no association was observed between CSH and underlying diseases, such as hypertension, diabetes and coronary artery disease (Table 2).

**Table 2:** Distribution of responses to carotid sinus massage by age, sex, and underlying diseases, such as hypertension, diabetes and coronary artery disease.

Variable	Response to CSM						TOTAL	p
	No CSH		Cardioinhibitory		Vasodepressor			
	n	%	n	%	n	%	n	
**Age**								**0.356^#^**
50-59	22	78.5	5	17.9	1	3.5	28	
60-69	30	69.7	9	20.9	4	9.3	43	
70-79	31	56.3	15	27.3	9	16.3	55	
≥ 80	24	61.5	12	30.8	3	7.6	39	
**Sex**								< 0.001*
Male	30	46.1	28	43.1	6	9.2	65	
Female	77	77.0	13	13.0	11	11.0	100	
**Hypertension**								**0.849^#^**
-	25	65.7	10	26.3	3	7.8	38	
+	82	64.5	31	24.4	14	11.0	127	
**Diabetes**								**0.095^#^**
-	90	68.1	28	21.2	14	10.6	132	
+	17	51.5	13	39.4	3	9.0	33	
**Coronary artery disease**								**0.401^#^**
-	99	66.4	35	23.5	15	10.0	149	
+	8	50.0	6	37.5	2	12.5	16	
Total	103	62	41	25	21	13	165	

CSM: carotid sinus massage; CSH: carotid sinus hypersensitivity;

# likelihood ratio test;

* chi-square test

**Figure 3:**
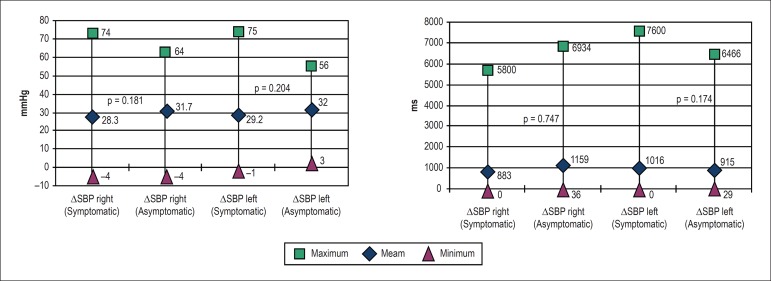
Magnitudes of systolic blood pressure response (ΔSBP) (above) and heart rate response (ΔRR) (below) in the symptomatic and asymptomatic groups during carotid sinus massage.

There was no difference (Figure 3) in the response to CSM when comparing the decrease in SBP (ΔSBP) and heart rate (ΔRR) between the symptomatic and asymptomatic groups. All patients were in sinus rhythm except for 2 individuals from the symptomatic group, who had atrial fibrillation (AF). One patient had persistent AF, and the other had paroxysmal AF.

During the maneuvers, 45 (45.45%) symptomatic patients and 34 (51.5%) asymptomatic patients dropped their SBP to values ≤ 85 mmHg. The proportions of patients who achieved SBP ≤ 85 mmHg in the series of CSM are shown in Table 3. The VD reflex increased from 8.0% to 31.3% in the symptomatic group and from 13.6% to 28.7% in the asymptomatic group, when applying the cut-off value of SBP ≤ 85 mmHg for the diagnosis of CSH, compared to the classical blood pressure criteria with a fall in SBP ≥ 50 mmHg. Therefore, the change in the cut-off value increased the diagnosis of CSH by 21.2% (or total 53.5%) and 15.2% (total 54.5%) in the symptomatic and asymptomatic groups, respectively.

**Table 3:** Proportions of patients with systolic blood pressure (SBP) ≤ 85 mmHg in the series of carotid sinus massage (CSM).

	Minimum SBP ≤ 85 mmHg during CSM				
	Right CSM 1 n (%)	Right CSM 2 n (%)	Left CSM 1 n (%)	Left CSM 2 n (%)	Total n (%)
Asymptomatic	24 (36.3)	24 (36.3)	20 (30.3)	16 (30.3)	66 (100)
Symptomatic	33 (33.3)	34 (34.3)	26 (26.2)	29 (29.2)	99 (100)

Although abnormal responses were similar in both groups, symptomatic patients reported more symptoms during CSM (41.4% vs. 27.3%, p = 0.063). The reported symptoms ranged from mild discomfort to syncope. In the symptomatic group, 20 patients reported presyncope, 16 patients reported dizziness, and 3 patients reported nonspecific symptoms. In the asymptomatic group, 5 patients reported presyncope, 10 patients, dizziness, and 2 patients, nonspecific symptoms. Only 2 patients in the symptomatic group had syncope, which occurred with ventricular pauses of 8.2 and 8.1 seconds. Symptoms reported with normal or abnormal responses made up 17.8% of normal, 78% of CI, and 47.1% of VD responders. Likewise, many asymptomatic patients showed a positive response without related symptoms, especially VD response (82.2% of normal, 22% of CI, and 52.9% of VD). Symptoms resulting from the CSM occurred mainly when the SBP dropped below 90 mmHg and/or the RR intervals extended longer than 2500 ms (Table 4).

**Table 4:** Correlation between occurrence of symptoms during carotid sinus massage and the value of minimum systolic blood pressure (SBP) and maximum RR interval obtained during the massage

	Symptoms	Mean ± SD	Median	Minimum	Maximum	n	p
Minimum right SBP (mmHg)	asymptomatic	102.5 ± 12.9	101	59	180	106	< 0.001*
	symptomatic	86.4 ± 23.6	85	42	151	59	
	Total	96.7 ± 23.7	96	42	180	165	
Minimum left SBP (mmHg)	asymptomatic	101.8 ± 20.7	98	64	185	106	< 0.001*
	symptomatic	89.0 ± 20.3	87,5	51	178	58	
	Total	97.3 ± 21.4	95	51	185	164	
Maximum right RR interval (ms)	asymptomatic	1326 ± 768	1154	625	5455	106	< 0.000#
	symptomatic	2639 ± 1762	1800	880	7500	59	
	Total	1795 ± 1369	1225	625	7500	165	
Maximum left RR interval (ms)	asymptomatic	1238 ± 564	1111	6326	4520	106	< 0.000#
	symptomatic	2772 ± 1891	1840	811	8160	59	
	Total	1786 ± 1419	1200	632	8160	165	

SD:standard deviation;

* Student's t Test;

# Mann-Whitney test.

The immediate reproducibility of the CSM response was evaluated by repeating the CSM during the same procedure. The heart rate response reproducibility was slightly superior as compared to the blood pressure response, with intraclass correlation coefficients of 0.68 for the right ΔSBP, 0.71 for the left ΔSBP, 0.83 for the right ΔRR, and 0.81 for the left ΔRR. The heart rate data demonstrate acceptable levels of conformity (above 0.75). Reproducibility of the abnormal blood pressure response (VD CSH) was observed in 40.8% (20/49 cases), and the abnormal heart rate response (CI CSH) in 48.5% (50/103 cases).

## Discussion

The diagnosis and management of syncope are still a challenging task in medical practice. In elderly patients, identifying the underlying diagnosis may be more complex due to multiple comorbidities, atypical presentations, amnesia from loss of consciousness, and difficulties in remembering and characterizing the episode.

The occurrence of OH is an important risk factor for falls and syncope, especially in the elderly, with 18.2% of prevalence.^20-23^ In this study, we observed more than twice the prevalence (29.2% vs. 12.1%) of OH in the symptomatic patients compared to the asymptomatic patients. This finding confirms the importance of investigating OH in aged patients with syncope, reinforcing OH as one of the most frequent causes of syncope in the elderly.

Differently from the results observed in the search of OH, similar responses were obtained during CSM in symptomatic and asymptomatic groups. This finding perhaps reinforces the hypotheses that CSH is not a diagnostic marker of a clinical syndrome. With a similar proposal to assess the prevalence of CSH and the diagnostic value of CSM, Tan et al.^24^ have found altered responses in 25% of the patients referred for evaluation of syncope and unexplained falls. This prevalence of CSH was lower when compared to the prevalence in another report^25^ in individuals older than 65 years, randomly sampled from an unselected community. In that study, the authors observed CSH in 39% of the patients, and, in a subgroup of patients with no history of syncope or falling, 35% had a hypersensitive response to CSM, and 36% had CSM-related symptoms. Thus, a positive test for CSH may not necessarily determine the cause of fainting, leaving the clinician with the difficult decision whether to accept the test as a confirmation of the cause of syncope, which sometimes might induce an incorrect diagnosis.

Solari et al.^26^ have proposed a cut-off value of symptomatic SBP ≤ 85 mmHg as more appropriate to identify the VD form of CSH in a study with 164 patients with CSM who produced spontaneous symptoms in the presence of hypotension or bradycardia (Method of Symptoms), or diagnosis of carotid sinus syndrome. The method does not require any cut-off value of asystolic pause or of the SBP fall induced by CSM, as positivity of the test is based on the reproduction of symptoms. They concluded that one third of patients with isolated VD form could not be identified by the classical blood pressure criteria for the diagnosis of CSH (a fall in SBP ≥ 50 mmHg), as compared with the ≤ 85 mmHg SBP cut-off value. Therefore, they offered this standardized objective methodology of classification of the VD reflex component to be used in clinical practice.^26^ Few large-scale studies have evaluated the diagnostic value of CSM. When positive, it suggests a tendency or predisposition to carotid sinus syndrome; however, this does not establish it as the cause of the patient’s syncope, with no “ideal” protocol, given that there is an inexorable trade-off between sensitivity and specificity without a “gold standard” test to prospectively validate it in populations with rigorously defined carotid sinus syndrome. Likewise, the reproduction of spontaneous symptoms to confirm the diagnosis as recommended by the European Society of Cardiology with the Method of Symptoms may be imprecise in this population, since prodromal symptoms are absent in up to 93% of patients with carotid sinus syndrome, and most of all with frequent memory and cognitive deficit, confounding the correlation. Additionally, any etiology that causes hypotension might result in symptoms similar to those determined by CSH, with the first symptoms of retinal and cerebral hypoperfusion expected in the upright position when SBP drops below 80 mmHg. An association between impaired cerebral autoregulation and the symptomatic presentation of CSH was demonstrated by Tan et al.^27^ in a study using transcranial Doppler ultrasonography during lower body negative pressure-induced systemic hypotension.^27^ They have demonstrated that individuals with symptomatic CSH have lower cerebral blood flow than do asymptomatic individuals with CSH in response to comparable reductions in systemic blood pressure, and have suggested that symptomatic individuals have an increased susceptibility to syncope or falls compared with individuals with asymptomatic CSH due to a lower ability to maintain cerebral blood flow in the face of a hypotensive challenge.

In our study, we observed that symptoms resulting from the CSM occurred mainly when the SBP dropped below 90 mmHg and/or the RR intervals extended longer than 2500 ms, regardless of the diagnosis associated with CSM. Associated with this factor, CSH is elicited by manual massage, which is a highly variable stimulus. This may be the reason for the low reproducibility of the positive response, as shown in this study.

While CSH has been observed in patients with syncope, and the symptoms were reproduced during CSM, there are no reports demonstrating that the hemodynamic alterations seen in the laboratory occur in a spontaneous event. Trying to establish the relationship between CSH and falls or syncopes, Schoon et al. have tested the hypothesis that head turning triggers hypotensive episodes in elderly with CSH. They have concluded that head turning may cause hypotensive episodes in the elderly. Head turning led to hypotension in 39% (total of 96 patients) of patients, with a mean SBP drop of 36 mm Hg (SD ± 13; range 20-76) with similar occurrence compared to healthy elderly, with 44% (total of 25 patients) and a mean SBP drop of 35 mmHg (SD ± 19; range 20-85). A drawback of the observational design is that it does not allow for conclusions about the causal relationships among head turning-triggered hypotension and syncope, and falls. They have also found a discrepancy between the occurrence of that head turning-triggered hypotension and related symptoms.^28^ Thus, the positive correlation between CSH and syncope and/or falls still needs to be redefined due to the accumulating evidence that CSM causes a similar positive response in the asymptomatic population with the current criteria to diagnose CSH. The cut-off value of symptomatic SBP ≤ 85 mmHg to identify the VD form of CSH may cause overdiagnosis, sometimes leads to misdiagnosis, with no benefits in treatment plus potential side effects outweighing the benefits. Other options, such as long-lasting ECG monitoring with documentation of spontaneous events, are the only way to corroborate the diagnosis and its correlation with laboratorial findings.

## Conclusion

In conclusion, no differences in the response to CSM were demonstrated between patients with and without syncope or presyncope. Carotid sinus hypersensitivity may be an unspecific condition in the evaluation of syncope. The best cut-off values of the asystolic pause and SBP based or not on the reproduction of symptoms are still a challenging task in medical practice. Consequently, clinical correlation and other methods of evaluation, such as long-lasting ECG monitoring, may be necessary to confirm CSH as a cause of syncope.

### Study limitations

The control group was composed of not completely healthy individuals, but with no significant heart disease and in stable clinical condition. It is already known that elderly people have an average of three comorbidities per person. Asymptomatic patients in this study were recruited from an outpatient geriatric unit. The institution is a referral tertiary cardiology center, and the patients usually have substantial clinical complexity. Even with the exclusion criteria, which led to the inclusion of only patients without significant heart disease and in stable clinical conditions at the time of selection, we observed that more patients with diabetes and coronary artery disease were in the asymptomatic group. On the other hand, patients in the asymptomatic group were a little older than those in the symptomatic group, with a mean age of 73.0 and 69.6 years, respectively. Despite this difference, patients in both groups are representative of the elderly population, in whom a positive vagal maneuver is believed to define the etiologic diagnosis of syncope. The presence of systemic underlying comorbidities in the asymptomatic group may be an important concern. The advanced age and the presence of simultaneous underlying diseases in these patients reinforce the hypothesis that CSH could be not much more than a laboratory finding related to aging and vascular diseases. We recognize that the difference in age and in comorbidities between groups could constitute a bias, but we are sure that both groups are representative of the elderly population in which unexplained syncope is a great challenge.

In this study the CSM was performed with the patient in the 70° upright position after 5 minutes in the orthostatic position different from other studies performed in the supine position. Thus, our findings may be different and, therefore, could not be applied to the CSM in supine position. We chose the orthostatic position because it is the most sensitive to detect CSH according to the study performed by Parry et al.,^29^ who have demonstrated that the specificity and sensitivity of the initially supine positive test were thus 74% and 100%, respectively, while the upright positive test had 100% specificity and sensitivity. For this reason, we performed CSM only in the orthostatic position in this study.
